# Heteroatoms (B, N, S) doped quantum dots as potential drug delivery system for isoniazid: insight from DFT, NCI, and QTAIM

**DOI:** 10.1016/j.heliyon.2022.e12599

**Published:** 2022-12-24

**Authors:** Henry O. Edet, Hitler Louis, Terkumbur E. Gber, Precious S. Idante, ThankGod C. Egemonye, Providence B. Ashishie, Emmanuella E. Oyo-Ita, Innocent Benjamin, Adedapo S. Adeyinka

**Affiliations:** a Computational and Bio-Simulation Research Group, University of Calabar, Calabar, Nigeria; b Department of Biochemistry, University of Cross River, Nigeria; c Department of Pure and Applied Chemistry, Faculty of Physical Sciences, University of Calabar, Calabar, Nigeria; d Research Centre for Synthesis and Catalysis, Department of Chemical Sciences, University of Johannesburg, South Africa

**Keywords:** DFT, Drug delivery, Interaction, Isoniazid, Quantum dots

## Abstract

Toxicity in drug includes target toxicity, immune hypersensitivity and off target toxicity. Recently, advances in nanotechnology in the areas of drug delivery have help reduce toxicity and enhance drug solubility and deliver drugs to target sites more efficiently. In this study, we present a novel heteroatom functionalized quantum dot (QD-NBC and QD-NBS) as an effective drug delivery system for isoniazid. The said QD has been computationally modeled to assess its effectiveness in delivering isoniazid to desired target. Density functional theory (DFT) calculations were performed on the QD at the B3LYP/6−311+G(d, p) level to assess its stability through the natural bond orbital (NBO) calculations, and frontier molecular orbital (FMO) before and after interaction with isoniazid drug to understand any change in molecular behavior of the surface. Appropriate intermolecular interactions between the QD and the drug were computed through the Quantum theory of atoms in molecules (QTAIM) and Non-covalent interaction to assess the various binding mechanism and possible interactions resulting to the effective delivery of the drug target. To understand and accurately appraise the binding energy of adsorption, DFT calculations were performed with different functionals (B3LYP, CAM-B3LYP, PBEPBE, GD3BJ & WB97XD/6−311+G (d, p)). The results from DFT calculations point the functionalized QDs to be stable with appreciable energy gap suitable for delivery purposes. The adsorption energy of the drug target with the QD is in the range of −24.73 to 33.75 kcal/mol which indicates substantial interaction of the drug with the QD surface. This absorption energy is comparable with several reported literature and thus prompt the suitability of the surface for isoniazid delivery.

## 1 Introduction

Drug delivery discusses strategies involved in transporting a combination of drugs to their intended destination to achieve the desired treatment effect. Recent advances in nanotechnology of drug delivery systems have impacted the medical field, these drug delivery systems help reduce drug toxicity [1]. However, drug delivery into living systems has been accomplished using nanoparticles like liposomes and dendrimers containing quantum dots. [2], amid other types of nanomaterials, fullerenes, carbon nanotube and quantum dots have concerned interest for medical use because of their distinct structural properties [3]. Machine learning (ML) is also a growing field with various applications which offers solutions to complex programs with the aid of computational methods [4]. ML uses digitized form of chemical structures which are experimentally and theoretically calculated for different approach and applications [5]. This approach provides insights for hidden data, which could be useful in the area of drug delivery [6]. Machine learning has enabled great advances in healthcare and pharmaceutical sectors.

Major advances in the use of machine learning include the development of new medications and therapeutic targets as well as improved diagnosis. The drug delivery system is stable thanks to machine learning techniques, which also anticipate drug dosage and increase drug permeability through bodily barriers [7]. Biocompatibility and less toxicity are an advantage for quantum dots in application for drug delivery system [8]. Density functional theory has been used to analyzed interactions between drugs and quantum dots, the DFT method B3LYP/6−311+G (d) was used to carry out optimization for quantum dots, a similar basis set for DFT B3LYP was used by MortezaVatanparast & Zahra Shariatinia for graphene quantum dot [9]. The values for Energy gap (isolated and interacted states), adsorption energy and thermochemical properties, quantum theory of atom in molecules, as well as the non-covalent interactions were used alongside the graph for density of states to determine the nature of interactions.

**Figure 1:**
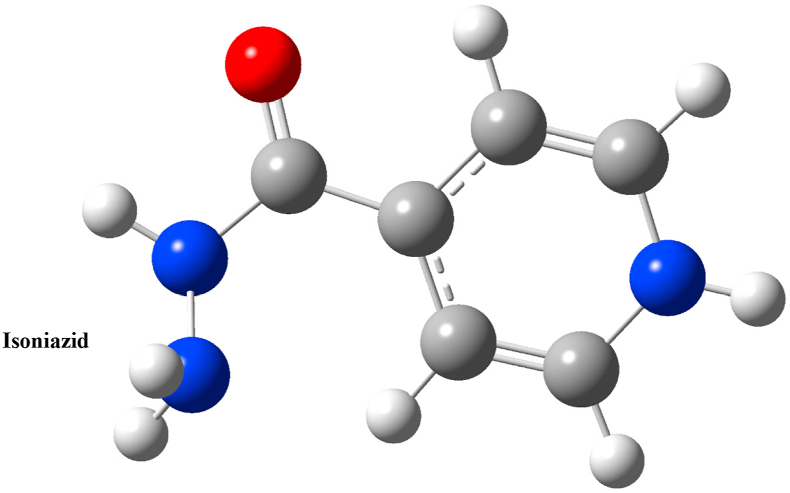
Optimized structure of isoniazid.

Recently, the usefulness and importance of nanocages and quantum dots was emphasized by Celaya [10]. The effectiveness of quantum dots was also highlighted by vatanparast [9]. Nanocarriers are said to reduce the therapeutic effect of drugs and improve their effectiveness [11]. In addition different nanotubes, fullerene and quantum dots have been used to deliver drugs to aim site with less toxicity and larger surface to volume ratio [11]. Previous research shows that Boron (B) and Nitrogen (N) doped nanostructures are non-cytotoxic and has several biological applications [12]. Saikia *et al.* have studied the structure and electronic properties of the noncovalent functionalization of BN nanotubes with isoniazid [13]. Furthermore, Judge and coworkers have synthesized a C60/isoniazid conjugate and successfully tested it for antimycobacterial activity [14]. Density functional theory (DFT) simulations were used by Mehrnoosh *et al.* (2017) to examine the possibility of C30B15N15 heterofullerene as a drug carrier for the anti-tubercular medication isoniazid [15]. The choice of B, S & N for doping on quantum dots is due to the wide considerations of heteroatom that moderates graphene properties [16]. Different functional were used to calculate the adsorption energy for accuracy and proficiency and was compared to previous work. Al, P and N doped graphenes have been studied.A contagious illness that affects the lungs and other body organs is tuberculosis. According to reports, approximately 10 million individuals will have had tuberculosis (TB) in 2020, which will cause 1.5 million deaths and rank as the second highest cause of COVID-19 death. [17, 18], as the initial anti-TB medication with potent antibacterial action, isoniazid, which is commonly used in other tuberculosis cases, is frequently advised. Although isoniazid works, it also has harmful side effects, including a rise in blood levels of liver enzymes and numbness in the hands and feet. Delivering isoniazid via nanocarriers will help minimize these unwanted effects [19]. In this study, in line with other research work, quantum dot surface was doped in other to enhance the sensitivity and delivery properties of isoniazid drug whose toxic level could result to severe health issues. Therefore, the purpose of this work is to study the potential of doped quantum dot surface as a nanocarrier or delivery agent for isoniazid. Herein, the investigation of heteroatoms; (Nitrogen, Boron and Sulfur) doped quantum dots, which are represented as (QD-NBC, QD-NBS) is reported. Electronic properties such as quantum descriptors, adsorption energy, thermodynamic properties, and adsorption distance were calculated and analyzed. Adsorption energy for isoniazid drug and doped quantum dots surface were calculated with different functionals B3LYP, CAM-B3LYP, PBE0, GD3BJ& WB97XD/6−311+G(d,p) in different from other studies. The nature of interactions was determined by quantum theory of atom in molecules (QTAIM) and non-covalent interactions (NCI). The studied drug molecule is presented in Figure 1.

.

## 2 Computational details

Density functional theory and geometrical optimization for the quantum dots and isoniazid drug was -achieved with the Gaussian16 and GaussView6.0.16 package [20, 21]. Natural bond orbital (NBO) 7.0 program was used to study inter molecular charge transfer as well as the stability of the surfaces [22]. The geometry optimization was achieved using the Becke’s three parameter exchange-functional combined with corrected correlation Lee, Yang and Parr functional (B3LYP) methods using 6−311+G(d,p) basis set. The optimized structures are in local minima on their potential energy surfaces, which are recognized by the absence of imaginary frequency in the Hessian matrix. The nature of interaction between the drug molecule and surface were attained using the quantum theory of atom in molecules (QTAIM) with the aid of multiwfn Analyzer [23]. Non-covalent interactions were achieved using RDG scattered, while the visualization was done using VMD [24], this was done to achieve intermolecular and van der Waals interactions, which aid in understanding the mechanism of isoniazid drug delivery by the doped quantum dot surface. In achieving the doped quantum dots surface, quantum dot surface which comprises of 36 carbon atoms (QD-CCC), Heteroatoms (B, N, S) replace carbon atoms in the central ring of the surface. To determine the adsorption energy, quantum dots and isoniazid were optimized with same basis set and was calculated using;

(1) Adsorption energy = ΔE_ads_ = E_(Drug/quantum dot)_ − (E_(Drug)_ + E _(Quantum dot)_)

The dispersion correction and induction moments interactions are the main constituents of the attractive vdW interaction at long ranges. Although, the overlap and exchange interactions also contribute at the short range repulsive regions [25]. The conventional pure-GGA and hybrid-GGA are the most popular DFT functionals which gives point by point clarification of both the ionic and the covalent interactions, but such methods cannot predict vdW interactions precisely, because they failed in calculating dispersion forces [26]. In which Becke 88 GGA functional is used for exchange and the LYP functional for non-local correlation. There are different approaches to account for dispersion interactions in DFT [27], from which DFT-GD3BJ dispersion correction method is a robust and widely used method in which dispersion interaction implemented as an add-on to standard Kohn–Sham density functional. The Becke 3-parameter Lee–Yang–Parr functional with dispersion correction B3LYP-GD3BJ, the long-range dispersion correction functional by the Head-Gordon group (ωB97XD), The new hybrid exchange correlation functional CAM-B3LYP which combines the hybrid qualities and the long range correction. The hybrid-exchange correlation (PBE0) all with the 6−311G (d) basis set were employed to study the interactions between the drug molecule and the doped graphene surface. Selected functional are expected to predict accurately weak interactions.

## 3 Results and discussion

### 3.1 HOMO-LUMO analysis

#### 3.1.1 Before drug delivery

Frontier molecular orbital is a theory that helps explain the use of HOMO-LUMO energy analysis within the system under investigation. HOMO-LUMO (Highest occupied and lowest unoccupied molecular orbitals) represents the ability to donate or receive electrons respectively to any surfaces and play an important role in its electrical and optical properties and chemical reactions and are represented in negative form (see Eqs. (2) and (3)). Large and small HOMO-LUMO spaces mean molecules that are “heavy” and “soft” chemically. If not, it was found that complex chemical regeneration is also related to its “hard” molecules with a lower HOMO-LUMO gap. HUMO/LUMO assists in calculating energy gap, chemical strength, chemical hardness, chemical softness and electrophilicity.

(2) $$IP={-E}_{HOMO}$$

(3) $$EA={-E}_{LUMO}$$

Hence, the global reactivity descriptors could be computed using Eqs. (4), (5), (6), and (7) as suggested in literatures [28, 29].

**Figure 2:**
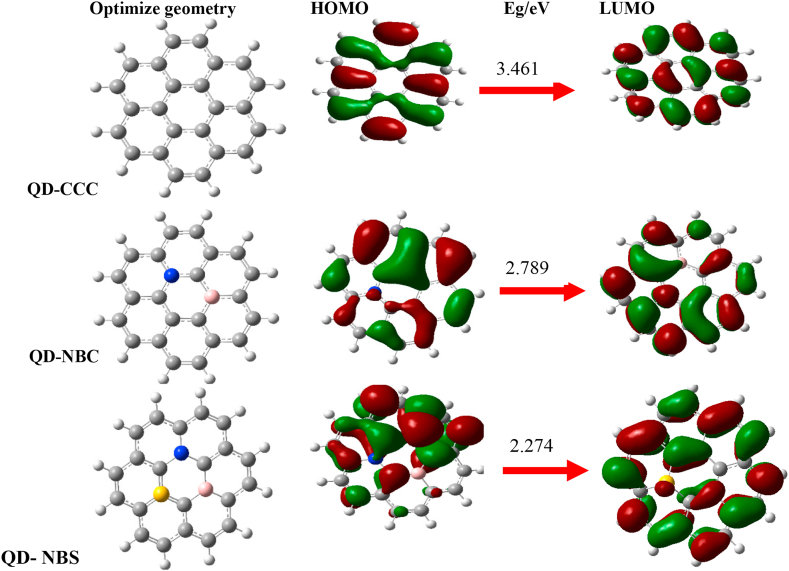
Optimized quantum dots, HOMO, and LUMO charge distributions.

(4) −μ=12(EHOMO+ELUMO)=χ

(5) η=12(IP−EA)=ELUMO−EHOMO2

(6) $$\omega =\frac{\mu^{2}}{2\eta }$$

(7) $$S=\frac{1}{2\eta }=\frac{1}{IP-EA}=\frac{1}{E_{LUMO}-E_{HOMO}}$$

where μ, χ, η, ω, and S are the chemical potential, electronegativity, chemical hardness, electrophilicity, and chemical softness respectively calculated as reported in Table 1 while the orbital density distributions are shown in Figure 2. In the isolated state, it is reported that QD-NBS is the most reactive surface due to it low energy gap and this makes it less stable this could be as a result of the doped atom which are highly electronegative (N and S). In contrast QD-CCC is less reactive but more stable due to it high energy gap, this is as a result of the electronegativity difference between C–C bond. When values for chemical hardness (η) are small it indicates great reactivity which is due to lower needed energy to promote an electron from the HOMO to LOMO orbitals. Obtained values for (η) ranges within 2.000–1.137 eV this suggest good surface reactivity. The polarizability of a compound which is determined by the value of chemical softness, this implies that the larger the value for chemical softness the higher the polarizability. From this work the values for chemical softness followed a trend of; QD-NBS 0.439 > QD-NBC 0.358 > QD-CCC 0.220. This describes that QD-NBS is the most polarized when compared to other surfaces. It is evident that QD-NBS is the most reactive and is strongly polarized. However, quantum dot surfaces exhibits potential for drug delivery due to the reactivity and stability level.

**Table 1:** Calculated HOMO-LUMO, Energy gap, Electrophilicity, chemical potential, chemical hardness and softness of studied quantum dots in isolated state.

Surfaces	HOMO/eV	LUMO/eV	Eg/eV	μ	Ω	η	*S*
QD-CCC	-5.72	-1.72	3.461	1.730	0.748	2.000	0.220
QD-NBC	-5.11	-2.32	2.789	1.394	0.697	1.394	0.358
QD-NBS	-4.61	-2.33	2.274	1.137	0.568	1.137	0.439

#### 3.1.2 After drug delivery

**Figure 3:**
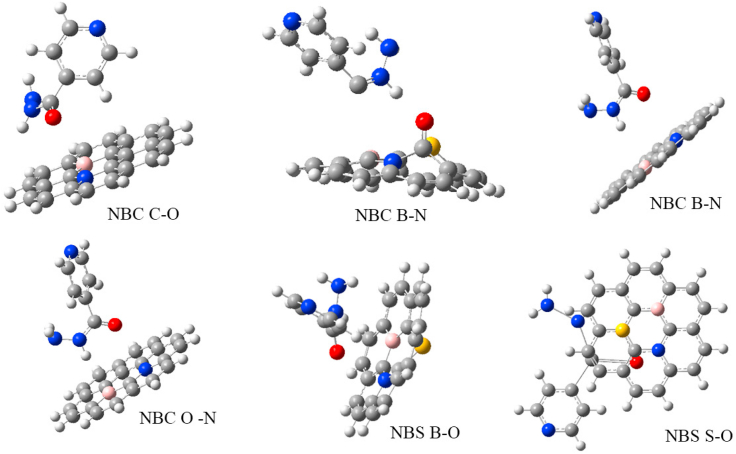
Showing interaction of isoniazid on QD-NBC, QD-NBS.

The HOMO-LUMO energy gap analyses of the interacted surfaces are presented in Table 2 while the various interaction modes between the quantum dots and the drug are displayed in Figure 3. From Table 2, QD-NBS was reported to be the most reactive due to its low energy gap in the isolated state. After interaction, it was observed that the interaction between sulfur and oxygen from the drug and the quantum dot NBS exhibited the same result as earlier reported. S–O interaction on QD-NBS has the lowest energy gap and makes it more reactive compared to other interactions. It is evident that sulfur reacts with nearly all other elements and gives a better interaction which makes it more reactive than other interactions. However, interactions between, B–N, B–O, N–O & C–O on NBC Quantum Dot exhibited high energy gap and makes it less reactive. From the result it can be concluded that QD-NBS (Isolated) & QD-NBS-S-O (After delivery) is the most reactive compared to others both in the isolated and interacted state.

**Table 2:** Calculated HOMO-LUMO and Energy gap values for various interactions.

Surfaces	HOMO/eV	LUMO/Ev	Eg/Ev
QD-NBC-B-N	-5.102	-2.207	2.895
QD-NBC-B-O	-5.102	-2.207	2.895
QD-NBC-N-O	-5.102	-2.207	2.895
QD-NBC-C-O	-5.243	-2.348	2.895
			
QD-NBS-B-O	-4.642	-2.272	2.370
QD-NBS-S-O	-5.421	-3.200	2.221

### 3.2 Natural bond orbital (NBO) analysis

Natural bond orbitals (NBO) provide details on how a compound’s donor and acceptor orbitals interact. The Lewis orbital type describes the bonding NBOs, whereas the non-Lewis orbital type describes the antibonding NBOs [30]. In NBO analysis, all potential interactions between filled and empty NBOs are taken into account, and their energy relevance is determined using second order perturbation theory [31]. Eq. (8) estimates the stabilization energy (E2) links with electron delocalization among Lewis (filled) and non-Lewis (unfilled) for each electron donor (i) and electron acceptor (j).

(8) $$E^{\left(2\right)}=q_{i\;}\frac{{\left(F_{i,j}\right)}^{2}}{\varepsilon_{j}-\varepsilon_{i}}$$

where *q*_*i*_ = donor orbital, *E*_*i*_ = diagonal element, *E*_*j*_ = orbital energies, *F*_(*ij*)_ = diagonal NBO fock matrix element. The degree of interaction between the donor and acceptor orbital is determined by the stabilization energy *E*^(2)^, the larger the stabilization energy the stronger the interaction [32]. Stabilization energy for the most interacting donor and acceptor orbitals in this study is presented in Table 3. Bonding to anti-bonding interactions were found to be the most common in the studied surfaces, which could be due to charge transfer occurring on the studied surface. The interactions observed between the donor and acceptor orbitals with transitions for QD-CCC are; σ(C_11_–C_12_) − σ∗(C_9_–C_10_), σ(C_11_–C_12_) − σ∗(C_7_–C_10_), σ(C_24_–C_25_) − σ∗(C_23_–C_24_), σ(C_9_–C_11_) − σ∗(C_7_–C_25_) and σ(C_10_– C_30_) - σ∗(C_2_–C_24_) with stabilization energies of 85,405.61 kcal/mol, 68,831.94 kcal/mol, 50,343.13 kcal/mol, 47,767.52 kcal/mol and 20,713.25 kcal/mol respectively. Also, for QD-NBC; σ(C_8_–B_36_) − Π(C_10_–C_19_**),** σ(C_8_–B_36_) − σ∗(C_21_–H_24_), σ(C_1_–H_17_) − σ∗(C_3_–C_4_), σ(C_1_–H_17_) − Π∗(C_2_–C_21_) and σ(C_7_–B_36_) − σ∗(C_28_–B_36_) with stabilization energies 17,824.67 kcal/mol, 15,417.45 kcal/mol, 5240.23 kcal/mol, 3042.52 kcal/mol and 747.11 kcal/mol respectively. Finally, transitions for the donor and the acceptor orbitals for QD-NBS are; σ(C_10_–C_11_) − σ∗(C_25_–C_26_), σ(C_4_–C_5_) − σ∗(C_25_–C_26_), σ(C_1_–H_16_) − σ∗(C_25_–C_26_), σ(C_11_–C_12_) − σ∗(C_13_–H_14_) and σ(C_8_–C_30_) − Π∗(C_22_–C_25_) with corresponding stabilization energies 13,994.76 kcal/mol, 13,762.25 kcal/mol, 12,713.99 kcal/mol, 2423.62 kcal/mol and 800.85 kcal/mol respectively. The greatest intermolecular and intramolecular interaction was found in QD-CCC; Donor σ (C_11_–C_12_) - Acceptor σ ∗(C_9_–C_10_) − (85,405.61 kcal/mol) resulting from C–C bonding interaction. In QD-NBS, the lowest noticeable interaction resulted from the interaction between (C_8_–C_30_) donor and σ∗(C_22_–C_25_) acceptor with an energy value of (800.85 kcal/mol). The major interactions observed in this study arise from the charge transfer between σ and σ∗ antibonding orbitals of carbon atoms as a result of an increase in electron density within the studied surfaces. The stability order of the examined QDs is as follows: QD-CCC>, QD-NBC >, QD-NBS.

**Table 3:** Second order perturbation theory analysis of the most interacting NBO’S of the studied quantum dots using B3LYP/6–311'+G (d) functional.

Compound	Donor(i)	Acceptor(j)	E(2) kcal/mol	E(j) − (i)	F(ij)
QD-CCC	σ(C_11_–C12)	σ∗(C_9_–C_10_)	85,405.61	435.45	172.301
	σ(C_11_–C12)	σ∗(C_7_–C_10_)	68,831.94	274.64	122.833
	σ(C_24_–C_25_)	σ∗(C_23_–C_24_)	50,343.13	628.81	531.986
	σ(C_9_–C_11_)	σ∗(C_7_–C_25_)	47,767.52	682.25	161.376
	σ(C_10_– C_30_)	σ∗(C_2_–C_24_)	20,713.25	190.21	120.01
QD-NBC	σ(C_8_–B_36_)	Π(C_10_–C_19_**)**	17,824.67	2.96	2.565
	σ(C_8_–B_36_)	σ∗(C_21_–H_24_)	15,417.45	3.60	7.030
	σ(C_1_–H_17_)	σ∗(C_3_–C_4_)	5240.23	0.60	1.590
	σ(C_1_–H_17_)	Π∗(C_2_–C_21_)	3042.52	0.28	0.829
	σ(C_7_–B_36_)	σ∗(C_28_–B_36_)	747.11	0.27	0.407
QD-NBS	σ(C_10_–C_11_)	σ∗(C_25_–C_26_)	13,994.76	16.52	13.621
	σ(C4–C5)	σ∗(C_25_–C_26_)	13,762.25	20.84	15.179
	σ(C_1_–H_16_)	σ∗(C_25_–C_26_)	12,713.99	31.25	17.834
	σ(C_11_–C_12_)	σ∗(C_13_–H_14_)	2423.62	23.09	14.427
	σ(C8–C_30_)	Π∗(C_22_–C_25_)	800.85	7.27	2.411

E^2^ = stabilization energy (kcal/mol).

E (i), E(j) = energy difference between donor and acceptors.

### 3.3 QTAIM analysis

QTAIM is a powerful tool for topology analysis containing the type and structure of bonds and intermolecular interactions. To further study the nature of interaction between the isoniazid drug and the different quantum dots QTAIM theory is employed to analyze the characteristics of the intermolecular hydrogen bond with the presence of critical points. The density of electrons ρ(r), Laplacian of electron density $\nabla^{2}\rho \left(r\right)$, Lagrangian kinetic energy G(r), Hamiltonian kinetic energy K(r), Energy density H(r) and Bond critical point (BCP) is explored using the quantum theory of atom in molecules [33]. QTAIM aids in interpreting bonding interactions and explicates the chemistry of interactions in simple terms. The significant and highest values for e(r), ∇e(r) G(r), K(r) and H(r) is presented in Table 4. The bond critical point (BCP), which indicates a critical point related to a bond or physical or chemical interaction, shows a strong critical point of interaction. Large values for ρ(r) indicate strong bond characteristics. From the results for the QD-NBS at B–O C_44_–O_45_ (0.587) bond between drug molecule and surface is said to be strong compared to other observed values for ρ(r). This interaction between the quantum dots with isoniazid had the highest electron density which could be as a result of the stability of the oxygen atom. This infers that oxygen aids the stabilization of the quantum dot and the drug during interaction, it’s expected that strong bonds exhibit high electron density as can be seen in the carbon – oxygen bond. The density of electrons ρ(r) at the BCP of coordinate bonds were observed to range from 0.235 to 0.587. Negative values for ∇^2^p(r) and H(r) indicate covalent interactions where the positive values of ∇^2^p(r) and negative values of H(r) indicate partially covalent interactions. Also, the values of ∇^2^p(r) > 0 and H(r) > 0 show weak interactions (non-covalent interactions). Bonds between O_48_–N_35_ for QD-NBC O–N are observed with negative values for ∇^2^p(r) (−0.810) and H(r) (−0.298) which indicates covalent nature for said interaction. However, for all interactions between drug molecule and surface values for ∇^2^p(r) > 0 and H(r) > 0 this show weak interactions and confirms non-covalent nature of interactions. Furthermore, the positive values for ∇^2^p(r) explains the energy of interaction, the positive values obtained for other interactions except for QD-NBC O–N. It is evident that the values across the interactions are similar and indicates stronger interactions between isoniazid drug and quantum dots NBC and NBS. The characteristics of interactions is defined by G(r)/V(r) values, where G(r)/V(r) < 0.5 is covalent, partially covalent when G(r)/V(r) > 0.5, and non-covalent when G(r)/V(r) > 1 respectively. For all interactions between drug molecule and quantum dot surface except for bond between S_53_–C_3_ in QD-NBS S–O with (0.728) values obtained for G(r)/V(r) are >1, this infers non-covalent nature of interactions and partially covalent nature. Results for intermolecular hydrogen interactions from QTAIM analysis suggest excellent correlation between the drug molecule and quantum dot surface.

**Table 4:** QTAIM values for Bond critical point, Density of all electrons, Laplacian electron density, Lagrangian kinetic energy, Hamiltonian kinetic energy and Energy density for studied quantum dots and isoniazid interactions.

Surfaces	Bond	ρ(r)	$\nabla^{2}\rho \left(r\right)$	G(r)	K(r)	V(r)	H(r)	G(r)/V(r)
QD-NBC B–N	N_49_– B_36_	0.493	0.169	0.320	-0.103	-0.216	0.103	1.481
	O_48_–N_35_	0.246	0.907	0.164	-0.625	-0.101	0.625	1.623
QD-NBC B–O	O_48_–N_35_	0.492	0.169	0.319	-0.103	-0.216	0.103	1.476
	N_49_–B_36_	0.517	0.175	0.388	-0.508	-0.377	0.508	1.029
QD-NBC C–O	N_51_–H_52_–C_2_	0.305	0.114	0.215	-0.697	-0.140	0.697	1.535
	N_49_–H_50_–C_22_	0.257	0.874	0.179	-0.388	-0.140	0.388	1.278
	C_47_–O_43_–C_6_	0.246	0.814	0.162	-0.412	-0.121	0.412	1.338
	N_49_–B_36_	0.265	0.813	0.161	-0.414	-0.120	0.414	1.341
	C_39_–H_44_–C_7_	0.243	0.860	0.158	-0.568	-0.101	0.568	1.564
QD-NBC O–N	N_49_– H_50_	0.493	0.169	0.320	-0.103	-0.216	0.103	1.481
	O_48_–N_35_	0.310	-0.810	0.984	0.298	-0.397	-0.298	2.478
QD-NBS B–O	C_8_–H_50_–N_46_	0.355	0.124	0.219	-0.904	-0.129	0.904	1.697
	C_35_–H_40_–C_1_	0.275	0.918	0.174	-0.553	-0.118	0.553	1.474
	C_44_–O_45_	0.587	0.198	0.466	0.495	-0.397	0.495	1.173
QD-NBS S–O	S_53_–C_3_	0.471	0.502	0.199	0.738	-0.273	-0.738	0.728
	N_46_– O_45_– O_3_	0.235	0.741	0.179	0.609	-0.173	0.609	1.034

### 3.4 Adsorption energies

To further evaluate the interactions between the isoniazid drug and the various quantum dots, adsorption energy (E_ad_) values were calculated for the optimized structures. The values for adsorption energy were calculated using Eq. (1). The adsorption energy for the studied systems were computed and presented in Table 5. The negative values for adsorption energy indicate higher contacts (attraction) and infer that adsorption process is exothermic, and also the results reflect successful drug-surface interactions. In comparison to NBC, doping the QD with N (NBS) increases the adsorption ability. NBC has adsorption energies of −24.29, 24.26, and 23.88 kcal/mol, while NBS has adsorption energies of 33.73 and 33.71 kcal/mol. Several computational methods have been used to calculate adsorption energies on various surfaces. For precision and stability, several functionals were utilized in computing adsorption energies for the examined interactions. Adsorption energies were computed, as shown in Table 6, using B3LYP, CAM-B3LYP, GD3BJ, PBE0 & WB97XD/6−311+G (d,p). The most stable complex is QD_NBS with adsorption energy of −33.75 kcal/mol at GD3BJ/6−311+G (d,p), this is due to the dispersion correction in functional which is suitable for predicting weak interactions. Herein, isoniazid interacted with QD_NBS through the different heteroatoms. The findings show that the isoniazid drug interacts successfully with quantum dots (NBC & NBS). Furthermore, the adsorption distance indicates the amount of interaction between the drug and the quantum dots. According to the findings, the adsorption distance between various interactions ranged from 2.817 to 4.246, indicating that the drug and quantum dots interact favorably.

**Table 5:** Adsorption energies for quantum dot surface and isoniazid drug.

Interacting surface	E_ads_(this work)
QD-NBC (B3LYP)	-24.29
QD-NBS (B3LYP)	-33.71
QD-NBC (PBE0)	-24.26
QD-NBS (PBE0)	-33.71
QD-NBC(CM-B3LYP)	-23.88
QD-NBS (CM-B3LYP)	-33.73
QD-NBC (WB97XD)	-24.28
QD-NBS (WB97XD)	-33.73
QD-NBC (GD3BJ)	-24.30
QD-NBS (GD3BJ)	-33.75

**Table 6:** Comparison of adsorption energies with different surfaces used for isoniazid drug delivery.

Comparative adsorption studies	Refs
-65.88	[9]
-30.61	[9]
-29.14	[9]
-47.29	[9]
-44.43	[9]
-21.96	[15]
-26.32	[38]
-22.45	[38]

#### 3.4.1 Comparison with other works

Our results for adsorption energy in comparison with other DFT calculations performed by Morteza and Vantaparast show that isoniazid interacts with AlN GQD from both –NH2 and –C=O sites [9]. Interestingly, a comparative study for the adsorption of isoniazid is presented in this session in comparison to our obtained results. Using B3LYP and M062x/6−31G (d,p) Morteza Vatanparast and Zahra Shariatinia [9] observed an adsorption energy of −65.88, −47.29, −44.43, −30.61, and −29.14 kcal/mol respectively for isoniazid on graphene. In addition, it was observed that the adsorption energies on the doped graphene surface are more negative than that of the pure GQD. Hence, doping increases the adsorption ability of surface. El-Mageed *et al.*, reported the adsorption energies of −26.32 and 22.45 kcal/mol for isoniazid delivered on Al_12_N_12_ nanocage [38]. Khodam Hazrati et al. reported that the adsorptions from −NH2 head group of isoniazid on the boron atoms are the most favorable interaction between isoniazid and C30B15N15 molecules with Eads of 21.96 kcal/mol [15]. Adsorption of isoniazid on a C30B15N15 surface shows that isoniazid drug has a greater tendency for adsorption on the QD-NBS in comparison to C30B15N15. Results from literature are in good agreement with that obtained.

### 3.5 Thermodynamic parameters

The enthalpy of a system (H), is the sum of its internal energy and the products of its volume and pressure, this is represented as ΔH = pΔv [33]. Enthalpy is a function for different measures in biological and chemical systems that help to distinguish between a system’s products and reactants. When ΔH > 0 the reaction is said to be endothermic and when ΔH < 0 the reaction is said to be the exothermic. In this study, the calculation for enthalpy of reaction for various quantum dots was performed using data attained from the optimized quantum dots using the following equations [34].

ΔH° (298K) = £_product_Δ_f_ H° prod (298k) − £_Reactants_ Δ_f_ H° react(298k)

(9) Δ _f_ H° (298k) = £ (E_0_ + H_Corr_) products − £ (E_0_ + H_corr_) reactants.

where E_0_ = Electronic energy; H_corr_ = Thermal correction to enthalpy.

In this study, results obtained for enthalpy of a reaction shows that the interaction between isoniazid and QD-NBC and QD-NBS resulted in negative value. QD-NBS has the highest enthalpy of reaction (−81.22 kcal/mol) and suggest the interaction is highly exothermic due to it negative value. Moreover, the enthalpy values obtained suggest a better interaction between the isoniazid and QD-NBC and QD-NBS.

The reversible function of work performed by a thermodynamic system is calculated using the Gibbs free energy (G) of the system, which is a thermodynamic potential. It expresses the whole of its temperature, entropy and enthalpy product [35]. It’s represented as ΔG^0^ = ΔH^0^ – TΔS^0^, where ΔG^0^ = Gibbs free energy, ΔH^0^ = change in enthalpy of the system, and TΔS^0^ = change in entropy of the system.

(10) Δ_r_ G^0^ (298k) = **Ʃ**(^Ɛ^0 + G_corr_) products – **Ʃ**(^Ɛ^0 + G_corr_) reactants

where; ^Ɛ^0 = Electronic energy; G_corr_ = Thermal correction to free energy.

Gibbs free energy (ΔG), is classified into spontaneous and non-spontaneous. High negative value for ΔG is an indication of a thermodynamically favored reaction. The calculated ΔG values in models A and B were slightly negative with values of −17.22 and −16.85 kcal/mol, respectively as reported by Gashemi *et al.* [36]. From our results obtained for ΔG compared to other works, the interactions between the isoniazid and the various quantum dots confirm that the interactions are highly spontaneous and thermodynamically favored as reported in Table 7. In addition, Isoniazid and QD-NBS gave the highest free energy of interactions (−81.22 kcal/mol) resulting with the highest negative value for ΔG. Therefore, Results obtained for thermodynamic properties is in good agreement with that for adsorption energy and confirms isoniazid drug delivery by quantum dot surface doped with heteroatoms to be thermodynamically favored.

**Table 7:** Thermodynamic properties for studied quantum dots and isoniazid interactions.

Parameters	Isoniazid	QD-NBC	[Isoniazid] [QD-NBC B–N]
^Ɛ^0	-472.798015	-925.278868	-1397.600735
^Ɛ^ZPE	0.143299	0.277170	0.410550
E_tot_	0.152633	0.291605	0.436350
H_corr_	0.153577	0.292549	0.437295
G_corr_	0.108020	0.237008	0.348949
^Ɛ^0 + ^Ɛ^ZPE	-472.762737	-925.001698	-1397.190186
^Ɛ^0 + E_tot_	-472.753402	-924.987263	-1397.164385
^Ɛ^0 + H_corr_	-472.752258	-924.986318	-1397.163441
^Ɛ^0+ G_corr_	-472.798015	-925.041859	-1397.251787
ΔG	-45.23		
ΔH	-45.23		
	Isoniazid	QD- NBC	[Isoniazid] [QD-NBC B–O]
^Ɛ^0	-472.798015	-925.278868	-1397.600735
^Ɛ^ZPE	0.143299	0.277170	0.410552
E_tot_	0.152633	0.291605	0.436352
H_corr_	0.153577	0.292549	0.437296
G_corr_	0.108020	0.237008	0.348969
^Ɛ^0 + ^Ɛ^ZPE	-472.762737	-925.001698	-1397.190183
^Ɛ^0 + E_tot_	-472.753402	-924.987263	-1397.164384
^Ɛ^0 + H_corr_	-472.752258	-924.986318	-1397.163489
^Ɛ^0+ G_corr_	-472.798015	-925.041859	-1397.251766
ΔG			
ΔH			
	Isoniazid	QD-NBC	[Isoniazid] [QD-NBC C–O]
^Ɛ^0	-472.798015	-925.278868	-1397.601737
^Ɛ^ZPE	0.143299	0.277170	0.410804
E_tot_	0.152633	0.291605	0.436459
H_corr_	0.153577	0.292549	0.437403
G_corr_	0.108020	0.237008	0.351489
^Ɛ^0 + ^Ɛ^ZPE	-472.762737	-925.001698	-1397.190934
^Ɛ^0 + E_tot_	-472.753402	-924.987263	-1397.165278
^Ɛ^0 + H_corr_	-472.752258	-924.986318	-1397.164334
^Ɛ^0+ G_corr_	-472.798015	-925.041859	-1397.250248
ΔG			
ΔH			
	Isoniazid	QD- NBC	[Isoniazid] [QD-NBC O–N]
^Ɛ^0	-472.798015	-925.278868	-1397.600735
^Ɛ^ZPE	0.143299	0.277170	0.410549
E_tot_	0.152633	0.291605	0.436350
H_corr_	0.153577	0.292549	0.437295
G_corr_	0.108020	0.237008	0.348941
^Ɛ^0 + ^Ɛ^ZPE	-472.762737	-925.001698	-1397.190186
^Ɛ^0 + E_tot_	-472.753402	-924.987263	-1397.164385
^Ɛ^0 + H_corr_	-472.752258	-924.986318	-1397.163441
^Ɛ^0+ G_corr_	-472.798015	-925.041859	1397.251794
ΔG			
ΔH			
	Isoniazid	QD-NBS	[Isoniazid] [QD-NBS B–O]
^Ɛ^0	-472.798015	-1285.210870	-1757.533716
^Ɛ^ZPE	0.143299	0.269131	0.402729
E_tot_	0.152633	0.284850	0.429701
H_corr_	0.153577	0.285794	0.430645
G_corr_	0.108020	0.227874	0.341943
^Ɛ^0 + ^Ɛ^ZPE	-472.762737	-1284.941740	-1757.130986
^Ɛ^0 + E_tot_	-472.753402	-1284.926021	-1757.104015
^Ɛ^0 + H_corr_	-472.752258	-1284.925076	-1757.103070
^Ɛ^0+ G_corr_	-472.798015	-1284.982997	-1757.191773
ΔG	-81.22		
ΔH	-81.22		
	Isoniazid	QD-NBS	[Isoniazid] [QD-NBS S–O]
^Ɛ^0	-472.798015	-1285.210870	1757.464601
^Ɛ^ZPE	0.143299	0.269131	0.400784
E_tot_	0.152633	0.284850	0.428014
H_corr_	0.153577	0.285794	0.428958
G_corr_	0.108020	0.227874	0.338648
^Ɛ^0 + ^Ɛ^ZPE	-472.762737	-1284.941740	-1757.063818
^Ɛ^0 + E_tot_	-472.753402	-1284.926021	-1757.036587
^Ɛ^0 + H_corr_	-472.752258	-1284.925076	-1757.035643
^Ɛ^0+ G_corr_	-472.798015	-1284.982997	-1757.125953
ΔG			
ΔH			

### 3.6 Noncovalent interaction (NCI)

Non-covalent interactions encompass the distinctions of interactions between molecules or within a molecule [37]. It is classified by; electrostatic interactions, van der Waal forces and hydrophobic effects. Non-covalent interaction is of critical importance in chemical and biological interactions and is heavily involved in biological processes in which large molecules binds explicitly to one another [38]. The most common approach to examining noncovalent interactions has been to assign van der Waals interactions (vdW), steric hindrance (SH), and hydrogen bonds (HBs). The results of QTAIM, in the previous section, described the nature of the interactions between isoniazid and quantum dots as non-covalent therefore, it is useful to confirm them by a non-covalent analysis. The two functions RDG and sign (λ_2_) (r)*p*(r) are used to define specific areas. Based on second eigenvalue of the electron density Hessian matrix (λ_2_) and electron density (ρ), sign(λ_2_)ρ < 0 represents an attractive interaction (such as hydrogen bonding); sign(λ_2_)ρ > 0 implies a repulsive interaction (such as steric repulsion); sign(λ_2_)ρ ≈ 0 indicates the presence of van der Wals (vdW) interaction [41]. Non-covalent interactions can be characterized in the region with low electron density and low RDG. The reduced density gradient (RDG) isosurface can be applied to visualization of weak interactions on a blue–green–red scale. RDG isosurface and VMD visualization for studied complexes is shown in Figure 3. RDG functions can be defined as;

(11) $$s=\frac{1}{2{\left(3\pi^{2}\right)}^{1/3}}\frac{\left|\nabla \rho \right|}{\rho^{4/3}}$$

**Figure 4:**
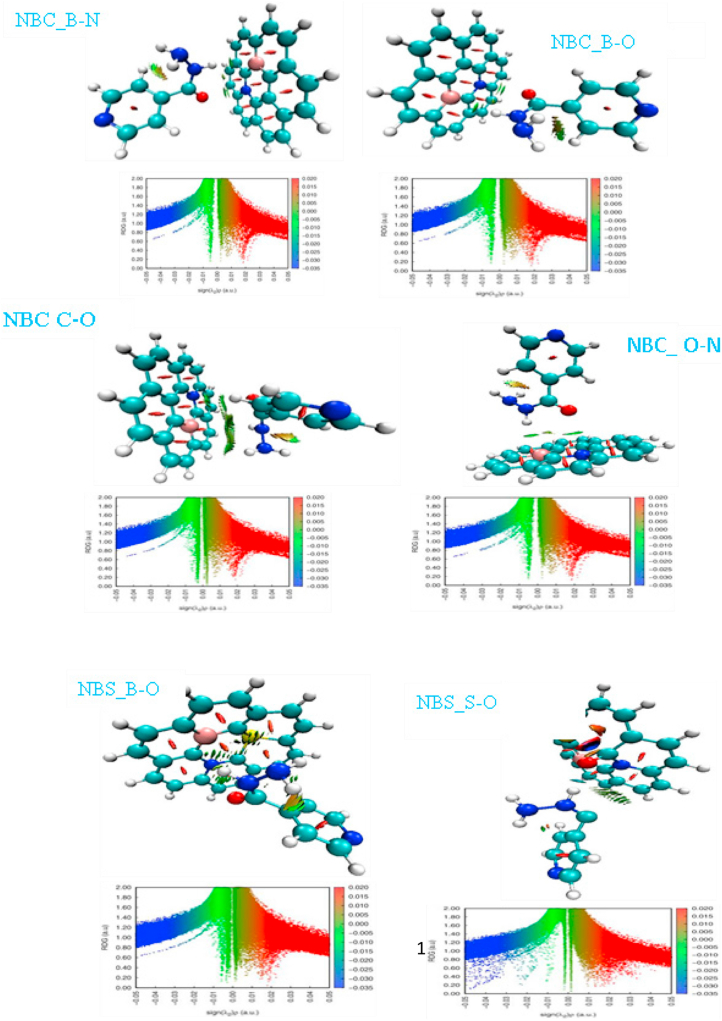
RDG isosurface and VMD visualization for studied quantum dots and isoniazid interactions.

The areas between the isoniazid drug and QD-NBC (B–N, B–O, C–O & O–N) are shown as green which infers a vdW interactions as shown in Figure 4. In addition, the blue region for isoniazid and QD-NBS S–O shows a hydrogen bond interaction with steric hindrances (Red), this could be as a result of hetero atoms nitrogen and sulfur replacing carbon atoms in the central ring of the quantum dots, and sulfur interacting from the quantum dot to oxygen from the drug. The green region between isoniazid drug and various interactions is the central driving force for isoniazid drug delivery in Figure 4 [9].

## 4 Conclusions

Wide-ranging theoretical investigations using DFT method for the interaction of isoniazid drug on NBS, NBC quantum dots was carried out to determine its potential for the delivery of isoniazid. Negative values for adsorption energy confirm surfaces are suitable for drug delivery. However, the highest negative value for adsorption was observed for QD-NBC and QD-NBS. The most stable complex for adsorption was observed for QD-NBC & NBS with GD3BJ/6−311+G(d,p) level. From the FMO section QD-NBS is the most reactive with small energy gap, both in the isolated and after delivery state. The negative values for enthalpy specify the exothermic nature of the isoniazid and quantum dots interaction. In addition, negative value for Gibbs free energy indicates spontaneous interaction between the isoniazid drug and quantum dots. The results obtained from non-covalent interactions suggest that the interaction between isoniazid and quantum dot NBC, NBS could be used as possible carriers for drug delivery application and is in agreement with the results for QTAIM analysis which revealed also the intermolecular interactions, these results indicate good relationship between the adsorption energy and thermodynamic properties calculated in this study. As a result we can conclude that quantum dots surfaces doped with heteroatoms can deliver isoniazid drugs.

## Declarations

### Author contribution statement

Henry O. Edet: Analyzed and interpreted the data; Wrote the paper.

Hitler Louis: Conceived and designed the experiments.

Terkumbur E. Gber, ThankGod C. Egemonye: Performed the experiments.

Precious Idante, Emmanuella Oyo-Ita: Analyzed and interpreted the data.

Providence B. Ashishie: Performed the experiments; Wrote the paper.

Innocent Benjamin: Contributed reagents, materials, analysis tools or data; Wrote the paper.

Adedapo S. Adeyinka: Contributed reagents, materials, analysis tools or data.

### Funding statement

This research did not receive any specific grant from funding agencies in the public, commercial, or not-for-profit sectors.

### Data availability statement

Data included in article/supplementary material/referenced in article.

### Declaration of interests statement

The authors declare no competing interests.

### Additional information

No additional information is available for this paper.
